# Near-Infrared Fluorescence Imaging for the Intraoperative Detection of Endometriosis: A Pilot Study

**DOI:** 10.3390/life12010015

**Published:** 2021-12-23

**Authors:** Mahdi Al-Taher, Jacqueline van den Bos, Ivon Terink, Sander van Kuijk, Nehalennia van Hanegem, Nicole Bouvy, Marlies Bongers, Laurents Stassen, Arianne Lim

**Affiliations:** 1 Department of Surgery, Maastricht University Medical Center, 6229 HX Maastricht, The Netherlands; j.vandenbos@maastrichtuniversity.nl (J.v.d.B.); n.bouvy@mumc.nl (N.B.); lps.stassen@mumc.nl (L.S.); 2 Department of Obstetrics and Gynecology, Maastricht University Medical Center, Research Institute GROW, 6229 HX Maastricht, The Netherlands; ivon_terink@hotmail.com (I.T.); lennie.van.hanegem@mumc.nl (N.v.H.); m.bongers@mumc.nl (M.B.); arianne.lim@mumc.nl (A.L.); 3 Department of Clinical Epidemiology and Medical Technology Assessment (KEMTA), Maastricht University Medical Center, 6229 HX Maastricht, The Netherlands; sander.van.kuijk@mumc.nl; 4 Department of Obstetrics & Gynecology, Maxima Medical Center, 5631 BM Veldhoven, The Netherlands

**Keywords:** endometriosis, near-infrared fluorescence imaging, indocyanine green, gynecology, laparoscopic surgery

## Abstract

Introduction: Endometriosis surgery is associated with a high risk of reoperation due to an insufficient recognition of endometriotic lesions. Our aim was to explore the role of near-infrared fluorescence (NIRF) imaging for the visualization and identification of endometriotic lesions next to conventional white light (WL) laparoscopy. Materials and methods: Fifteen women scheduled for diagnostic laparoscopy in whom peritoneal endometriosis was suspected were included. Peritoneal exploration was performed in WL, followed by NIRF imaging after ICG administration. Biopsies of all the suspected lesions were taken for histological examination. Subjective evaluations of the equipment and NIRF imaging were also performed. Results: Only 61% (44) of the biopsied lesions contained endometriosis. The positive predictive value (PPV) for the lesions found in WL was 64%. The PPV for the lesions found under NIRF was 69% and the PPV for the lesions found in both modes was 61%. The mean satisfaction of surgeons regarding the surgical procedure and equipment using both imaging modalities was 6.5 (*p* > 0.05) on a 10 item Likert scale and the mean satisfaction with the quality of the NIRF imaging was 7.4 (*p* > 0.05). Conclusion: In this study, the additional value of NIRF imaging, although feasible, was found to be limited for the intraoperative detection of endometriotic lesions.

## 1. Introduction

Endometriosis, defined as the presence of endometrial tissue outside the uterine cavity, is a disease affecting 10 to 15% of women in their reproductive age and up to 90% of women presenting with pelvic pain [1,2,3,4]. The development of endometriosis is a process in which the endometrial stromal cells acquire and lose parts of their cellular function in order to gain the ability to proliferate, migrate, and invade outside the uterine cavity [5]. The presence of such cells in the peritoneal cavity and what leads to the development of endometriosis is a complex process with a large number of interconnected factors, potentially both inherited and acquired [6].

Endometriosis reduces both physical and mental quality of life. Consequently, an effective treatment for endometriosis reduces patient discomfort and social burden [1,7,8,9,10,11]. 

The current gold standard of endometriosis diagnosis is to perform a diagnostic (conventional white light (WL)) laparoscopy combined with histological confirmation, as visual detection using WL alone is subjective and prone to misdiagnosis [12]. Treatment can be either conservative or surgical [13]. The surgical treatment of peritoneal endometriosis includes a diagnostic laparoscopy with excision or ablation of lesions. Laparoscopic excision is associated with overall pain reduction after 6 and 12 months and with increased ongoing pregnancy and live birth, as compared to diagnostic laparoscopy without the excision of lesions [14,15]. In the long term, laparoscopic local excision of endometriotic lesions is known to feature a high recurrence rate, involving pain, as well as a high risk of reoperation, which is estimated to range from 20 to 40% and which increases over time [16,17,18]. Incomplete detection and resection due to the variation in appearance of endometriotic lesions and the lack of adequate visualization [19] may be a potential explanation for the high recurrence rate. Several intraoperative imaging techniques have been described for the detection of endometriosis [20]. Near-infrared fluorescence imaging (NIRF) [21] is a promising technique for the enhanced detection of endometriotic lesions. This technique is based on the use of a fluorescent dye, which is either taken up by the tissue or transported intraluminally. It was also extensively studied for bile duct and bowel perfusion imaging, among others [22,23]. We hypothesize that this technique is useful during laparoscopic surgery to facilitate the detection of endometriosis by visualizing the differences in vascularization compared to adjacent tissue or via the direct uptake of the dye into the endometriotic tissue [21,24,25,26]. 

The aim of this study was to explore the feasibility of NIRF laparoscopy after intravenous indocyanine green (ICG) to visualize and identify endometriotic lesions. 

## 2. Materials and Methods

All the consecutive patients who visited the Department of Obstetrics and Gynecology of the Maastricht University Medical Centre (the Netherlands) and who were scheduled for an elective diagnostic laparoscopy due to a suspicion of peritoneal endometriosis were recruited for this study. The inclusion criteria were: women scheduled for elective laparoscopy for the evaluation of endometriosis, aged 18 years or older, premenopausal, and able to understand the nature and intent of the study. The exclusion criteria were the following: pregnancy or breast-feeding, known hypersensitivity/allergy to ICG or iodine, known hyperthyroidism or autonomic thyroid adenomas, a history of impaired liver or renal function, and an inability to provide written informed consent. 

### 2.1. Study Equipment and Fluorescent Dye 

A commercially available laparoscopic fluorescence imaging system (KARL STORZ GmbH & CO. KG, Tuttlingen, Germany) was used. The equipment included a plasma light guide and a 30°, 10 mm laparoscope applicable for NIRF imaging. Visual 2D recordings were made during the laparoscopic procedure. A 5 mg/mL ICG Verdye injection powder (Diagnostic Green GmbH, Aschheim, Germany) was prepared according to the manufacturer’s instructions.

### 2.2. Surgical Procedure

All the operative procedures were performed by two gynecologic surgeons (AL and NvH) with extensive experience in laparoscopic endometriosis surgery. After introducing the laparoscopic trocars, the abdominal cavity was systematically inspected for the presence of endometriotic lesions, first with WL, followed by NIRF imaging after intravenous ICG administration. 

An intravenous bolus of 2.5 mg of ICG was administered through a peripheral catheter in the arm of the patient by the anesthesiologist and dosing was repeated up to three times when deemed necessary by the operating surgeons. This dose is well below the maximum IV dose for ICG, which is 5 mg/kg of body weight. Repeated administration aims at repeated imaging of tissue vascularization. Imaging for this purpose should be performed within two minutes of administration due to the fast appearance and wash-out in case of perfusion imaging [27]. For all visualized lesions, the location, size, light mode, and aspect were described on the intraoperative registration form. Concerning ICG administration, the following parameters were collected: time of first ICG injection, time of first visual detection of fluorescence, and time of additional injection(s). Biopsies were taken from all the suspected endometriotic areas seen in both WL and NIRF mode, and from areas that appeared only in one of the modes. To serve as a negative control, one biopsy from a randomly chosen area without any visible or fluorescent lesions was taken per patient. The biopsies were assessed by a pathologist with extensive experience in endometriotic conditions. 

Postoperatively, the quality of imaging, satisfaction with the procedure and use of equipment were scored by the surgeons on a 10 point Likert scale. Likert scale responses are frequently used scoring schemes to attempt to quantify people’s opinions, interests or the perceived efficacy of an intervention [28]. In the current study, parameters including quality of WL and NIRF visualization, additional value of NIRF laparoscopy over WL imaging, and satisfaction with the procedure and use of the equipment in general were accorded scores from a minimum of 1 to a maximum of 10. One point corresponds to the lowest level of satisfaction and 10 points to the highest. Other registered parameters were additional remarks by the surgeon, other findings, and intraoperative complications, regardless of whether these were related to NIRF imaging. Postoperative care was performed in accordance with the usual care protocols. 

Due to the study characteristics, there was no blinding of surgeons and patients. The pathologist responsible for analyzing the biopsies was blinded to the light mode by which the lesion was identified, and whether or not the biopsy was taken from a suspected lesion or as a control peritoneal sample. The biopsies were numbered and named after their location, since the location of the biopsy is mandatory in the histological report. 

### 2.3. Statistical Analyses

Descriptive statistics were used for the patient and surgical procedure characteristics. They were also used to provide an insight into the surgeon’s satisfaction with the procedure and equipment. The number of endometriotic lesions found in total and per patient using WL and using WL combined with NIRF were reported. The positive predictive value (PPV) of both modalities used to detect the endometriotic lesions was calculated using histology as the gold standard. The PPV was defined as the number of correctly identified positive lesions for endometriosis divided by the total number of suspected lesions for endometriosis. The sensitivity could not be calculated as the total number of actual endometriotic lesions was unknown. Additionally, specificity could not be calculated since tissue that did not seem abnormal was not biopsied and could not be verified, except for a single control biopsy per patient. The only means to be correctly and surely informed of missed endometriotic lesions would involve a total peritonectomy to achieve a histological assessment. A *p* value of <0.05 was considered significant. SPSS software (IBM SPSS Statistics for Windows, Version 24.0. Armonk, NY, USA) was used to analyze and present the data. 

### 2.4. Ethical Approval

This single-center prospective feasibility study was approved (on 14 December 2016) by the Medical Ethics Committee of the Maastricht University Medical Centre (MUMC+) and was performed at this institute. The study protocol was registered with ClinicalTrials.gov (NCT03017989). 

## 3. Results

### 3.1. Patient Characteristics

Fifteen women with suspected peritoneal endometriosis and a scheduled diagnostic laparoscopy were prospectively enrolled in this study between January 2017 and December 2018. All the included women had a revised American Society for Reproductive Medicine (rASRM) classification of endometriosis stage I or stage II endometriosis [29]. 

The patient characteristics are summarized in Table 1. Three women had an American Society of Anesthesiologists (ASA) physical status score of 2 and 13 women had a score of 1. Three women had a history of prior abdominal surgery. 

The total time per procedure, defined as the time from the first incision until the removal of the laparoscope, varied from 26 to 110 (mean: 47) min. A total dose of 5.0 up to 7.5 mg of ICG dye was administered intraoperatively in boluses of 2.5 mg each. For all the procedures, NIRF imaging started within two minutes of dye administration (Table 2). 

**Figure 1:**
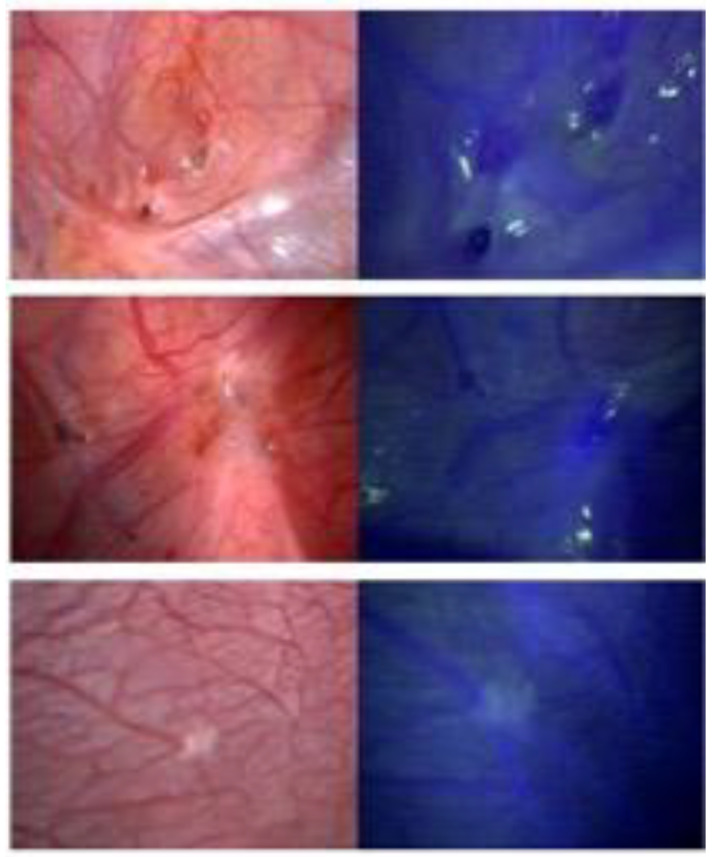
Intraoperative visualization of lesions, in which conventional white light imaging is shown to the left and near-infrared fluorescence imaging to the right.

In total, 87 biopsies were taken. Sixty-six lesions were identified during inspection in WL mode. NIRF mode confirmed 30 of these lesions and identified an additional 6 suspected lesions, which were not seen in WL mode. Fifteen biopsies served as negative control biopsies (Table 3). The number of biopsies taken per patient varied from 1 to 14 (mean: 5.6). A selection of the endometriotic lesions seen in WL and NIRF mode are presented in Figure 1. No complications or adverse events due to NIRF imaging were observed. 

### 3.2. Pathology Assessment of Biopsies

Of the 72 biopsies that were taken from suspicious lesions seen in either or a combination of imaging modes, 44 (61%) contained endometriosis. As a result, 28 biopsies (39%) that were macroscopically expected to contain endometriosis did not show endometriosis according to the pathologic assessment. All the random control biopsies were free from endometriosis (Table 4). 

The PPV for all the lesions found in WL mode was 64%. The PPV for lesions found under NIRF mode was 69%, and the PPV for lesions found in either mode was 61%. This difference was not statistically significant (*p* > 0.05). The PPV for WL only and for NIRF only is not provided as in clinical practice both imaging modalities are used in the same procedure. The calculation of the PPV is presented in Table 5. 

### 3.3. Surgeons’ Satisfaction

The surgeons’ satisfaction with the surgical procedure, equipment, and NIRF imaging is presented in Table 6. In this table, the overall general satisfaction of surgeons with the surgical procedure and equipment using both imaging modalities ranged from 2 to 9, with a mean of 6.5 (*p* > 0.05). Surgeons’ satisfaction with respect to the added value of NIRF over WL imaging only for the identification of endometriotic lesions ranged from 1 to 7, with a mean of 2.3 (*p* > 0.05). Satisfaction with the quality of WL laparoscopy ranged from 6 to 10, with a mean of 8.6 (*p* > 0.05), and satisfaction with the quality of NIRF imaging ranged from 5 to 9, with a mean of 7.4 (*p* > 0.05). 

## 4. Discussion

The primary objective of this pilot study was to investigate the feasibility of intraoperative NIRF imaging using ICG administration for the enhanced detection of endometriotic lesions during laparoscopic surgery. In clinical practice, the use of NIRF imaging would be complementary to conventional WL imaging, and as a result, it should not be regarded as a stand-alone technique for this purpose [31]. In this study, despite the addition of this novel imaging technique, a total of 39% (28) of the biopsied lesions were not endometriotic and could have been spared. The PPV for lesions found in WL mode was 64%. With the addition of NIRF imaging, the PPV increased to 69% (*p* > 0.05). The PPV for lesions found in either one or a combination of the modes was 61%. NIRF imaging helped to find two extra endometriotic lesions, which would have been missed. On the other hand, the imaging in NIRF mode only resulted in four biopsies, which were unnecessary. Using the combination of both imaging techniques, the biopsies that were meant to be negative controls turned out to be negative for endometriosis at pathologic examination. For ethical reasons, a limited number of such biopsies was taken. However, in this sample, this resulted in a negative predictive value (NPV) of 100%. 

Based on these results, the conclusion is that NIRF imaging, as applied in this study, may not be sufficient to identify endometriotic lesions intraoperatively, since 19 out of 44 histologically proven endometriotic lesions (48%) were missed with this imaging mode. However, if lesions were suspected in both imaging modes, the probability of these lesions really being endometriosis was increased compared to each of the imaging modes separately. In addition, through NIRF imaging, two additional endometriotic lesions were identified, which were missed under WL mode. This supports the potential additional value of NIRF imaging during laparoscopic surgery for endometriosis. 

Van Lier et al. [32] found that 43 out of 66 (65%) endometriotic lesions were not identified with fluorescent imaging. In this study, a bolus of 0.25 mg of ICG in a solution of 1 mg/mL was administered at the start of NIRF imaging, and imaging was continued for 5 min. A second bolus of 0.25 mg of ICG was administered when deemed necessary. A significantly decreased sensitivity rate was found compared to WL imaging. Potential explanations for this decreased sensitivity are the low dose of ICG administered and the short period of time (maximum of 5 min after dye administration) of NIRF imaging. 

In a prospective study by Cosentino et al. [26], the authors described 27 women in whom NIRF imaging using ICG showed a sensitivity of 82% and a specificity of 97.9%, confirming 75 of the 95 lesions (79%) identified as pathologic with WL imaging. This study shows a higher detection rate compared to our present study. As the authors state in their discussion, a possible explanation for these results is that their study included a higher percentage (~90%) of advanced-stage endometriosis (stages III and IV according to the revised American Society for Reproductive Medicine), which is typically not difficult to detect laparoscopically and may therefore have been easier to detect with NIRF imaging. Another difference between our study and that performed by Consentino et al. is that a significantly higher dose of ICG was administered (0.25 mg/kg) by this team and NIRF imaging started after an interval varying from a minimum of 5 to a maximum of 30 min. Consequently, the higher the stage of endometriosis, the higher the dose of ICG, and the longer time between ICG administration and NIRF imaging may have led to the more favorable results compared to our study. 

In a single-center prospective study by Siegenthaler et al., PPVs were found to be 89.8%, 68.8%, and 86.7% for WL laparoscopy alone, NIRF visualization alone, and the combination of WL + NIRF, respectively. In this study, an ICG dose of 0.3 mg/kg of body weight was used, which is also significantly higher than the dose administered in our study [33]. The authors concluded that NIRF imaging with ICG offered minimal additional value. However, increasing the ICG exposure time over 20 min led to a significantly positive effect on the detection rate of endometriotic lesions [33]. 

Unlike the aforementioned studies, we did not provide the sensitivity and/or specificity of this novel technique as this would assume that all peritoneal endometriotic lesions could be detected laparoscopically. Experience shows that laparoscopic resection of endometriotic lesions carries a risk of reoperation of 20 to 40% over time [19]. This can be partly due to the incomplete detection and removal of endometriotic lesions during the first surgery. We therefore assume that we have inevitably missed endometriotic lesions in our surgeries, with the consequence that we are not fully informed of the false negative rate and the total amount of positive lesions. 

In a prospective case series by Jayakumaran et al., NIRF imaging allowed to visualize a statistically significant higher number of lesions compared to that of robotic and laparoscopic WL [34]. In this study, an intraoperative single dose of 0.25 mg of ICG was administered intravenously prior to the systematic NIRF visualization of the abdominal cavity and search for lesions suspected for endometriosis. With robotic (da Vinci® Xi™ Surgical System) NIRF imaging, a significantly higher number of endometriotic lesions was found as compared to laparoscopic and robotic WL imaging. 

A limitation of the current study is that at the time when it was initiated, very limited clinical experiences had been published that described the potential benefits of NIRF imaging in the visual detection of peritoneal endometriosis resection surgery. Based on these studies with NIRF imaging and based on our experience with NIRF imaging in other fields of laparoscopic surgery, we hypothesized that the main working mechanism of NIRF imaging for endometriosis would be the imaging of the level of vascularization. Lesions can be hypervascularized, but also hypovascularized due to fibrosis. Mixed vascularization within a lesion can also be present. This explains our focus on NIRF imaging shortly after ICG administration. When ICG is retained in endometriotic tissue, a longer time interval between administration and assessment is warranted. As a result, we chose to administer an intravenous bolus of 2.5 mg of ICG intraoperatively and to repeat this protocol several times when deemed necessary by the operating surgeons [23]. However, based on the aforementioned studies, increasing the time interval between ICG administration and NIRF imaging would likely result in improved NIRF imaging of the lesions in comparison to our study. A potential explanation is the dye wash-out from healthy tissue over time (leading to a reduction in background fluorescence) and the retained ICG in endometriotic lesions due to vessel leakage in these hypervascularized tissues, a phenomenon that has been described previously [35]. 

The 10 item Likert scale used in this study is a tool that can be used assess the satisfaction and attitude of surgeons towards this new technique [28]. Despite the safety of the procedure and the overall acceptable satisfaction with the quality of the equipment and NIRF imaging in this study, the surgeons did not consider NIRF imaging to offer any significant added value during their laparoscopic procedures. This may have been influenced by the operative regimen, in which the first evaluation in each patient was performed in WL followed by NIRF imaging. Consequently, in most cases, the surgeons were already informed of the locations of the suspected endometriotic lesions and may have been influenced by this at the time of the NIRF imaging. Modern NIRF imaging systems with a so-called overlay mode, in which NIRF imaging is superimposed in real time onto the WL image, are promising tools to enhance visibility for surgeons without interfering with the surgical procedure and may increase the surgeon’s satisfaction with the technique. 

Another limitation of this study that needs to be addressed is the limited number of included patients, which prevents solid statistical conclusions from being drawn. 

In our study, as well as in previously discussed studies, the feasibility of NIRF imaging for endometriosis was shown. However, the dye concentration administered and the timing and duration of NIRF imaging can probably be improved for the enhanced identification of endometriotic lesions. This should be the focus of future studies. A potential strategy could be to administer a fixed amount of dye per kg of body weight prior to surgery with a waiting time of at least 30 min to the first NIRF assessment, followed by an additional bolus of ICG during surgery after this first assessment in order to identify any additional missed lesions. With this approach, it might be possible to improve the identification of both hypovascularized and hypervascularized lesions. Future studies should also evaluate learning curves for NIRF imaging, long-term outcomes such as disease-free intervals, recurrence rates, and quality of life, as well as the cost-effectiveness of this new technique. 

## 5. Conclusions

In this study, the additional value of NIRF imaging, although feasible, was found to be limited for the intraoperative detection of endometriotic lesions. Future research may investigate the ideal ICG timing and dosing, as well as the best operative regimen to maximize the enhancement of endometriosis detection using near-infrared fluorescence imaging during laparoscopy. 

## Figures and Tables

**Table 1:** Patient characteristics.

Patient No.	Age	ASA Score	BMI	Past Medical History
1	37	1	22.90	
2	36	1	23.90	
3	32	1	25.60	
4	29	1	20.34	
5	27	1	19.96	
6	32	1	33.65	Ileocecal resection for NET
7	19	1	19.35	
8	20	1	21.72	
9	39	1	27.68	
10	27	2	25.91	
11	35	1	28.08	
12	37	2	20.68	2× Rectopexy + Mesh
13	41	1	24.16	
14	35	2	24.50	2× Laparoscopy for endometriosis
15	32	1	21.00	Hepatic focal nodular hyperplasia

ASA: American Society of Anesthesiologists; BMI: Body Mass Index; NET: neuroendocrine tumor.

**Table 2:** Characteristics of the surgical procedure.

Patient No.	Laparoscopy Time (min)	Number of ICG Boluses (2.5 mg/bolus)	Total Amount of ICG (mg)	Time from ICG Administration to NIRF (min)
1	68	3	7.5	2
2	62	2	5	0.5
3	41	2	5	1
4	38	2	5	1
5	47	2	5	1
6	79	2	5	1
7	35	2	5	1
8	26	2	5	1
9	110	3	7.5	1
10	58	2	5	1
11	38	2	5	1.5
12	103	2	5	1
13	31	3	7.5	1
14	48	3	7.5	1
15	78	3	7.5	1

ICG: Indocyanine green; NIRF: near-infrared fluorescence.

**Table 3:** Biopsies taken under the two imaging modes.

Patient No.	Biopsies Taken (Total)	Biopsies of Suspected Lesions (WL Only)	Biopsies of Suspected Lesions (NIRF Only)	Biopsies of Suspected Lesions (Both Modes)	Control Biopsies
1	6	3	0	2	1
2	8	5	0	2	1
3	6	4	1	0	1
4	2	1	0	0	1
5	8	4	1	2	1
6	5	2	1	1	1
7	3	2	0	0	1
8	2	0	0	1	1
9	9	6	0	2	1
10	5	0	0	4	1
11	3	0	1	1	1
12	9	1	1	6	1
13	1	0	0	0	1
14	6	4	1	0	1
15	14	4	0	9	1
Total	87	36	6	30	15

WL: white light; NIRF: near-infrared fluorescence.

**Table 4:** Biopsy results per mode of visualization.

Variable	WL Mode		NIRF Only	Total
	WL Only	NIRF Mode		
		WL + NIRF		
Endometriosis	42		2	44
	19	25		
		23		
Non-endometriosis	24		4	28
	17	11		
		7		
Total	66		6	72
	36	36		
		30		

WL: white light; NIRF: near-infrared fluorescence.

**Table 5:** Calculation of the PPV for the (combination of) imaging modalities.

	WL and WL + NIRF	WL + NIRF and NIRF
Endometriosis	19 + 23 = 42	2 + 23 = 25
Non-endometriosis	17 + 7 = 24	4 + 7 = 11
Total number of lesions	36 + 30 = 66	6 + 30 = 36
PPV	42/66 = 64%	25/36 = 69%

PPV: positive predictive value; WL: white light; NIRF: near-infrared fluorescence.

**Table 6:** Subjective assessment of procedures by surgeons.

Patient No.	Equipment Satisfaction	Added Value of NIRF	Quality of NIRF	Quality of WL	
1	5	1	6	9	
2	8	1	8	10	
3	7	2	8	10	
4	7	1	8	10	
5	7	2	8	8	
6	8	2	8	9	
7	3	1	9	9	
8	8	1	8	9	
9	2	1	5	6	
10	9	1	7	9	
11	5	5	7	9	
12	7	6	8	7	
13	8	1	5	9	
14	7	7	8	7	
15	6	3	8	8	

WL: white light; NIRF: near-infrared fluorescence [30]. On this scale, a ‘’1’’ equals the lowest level of satisfaction and a ‘’10’’ equals the highest level of satisfaction.

## Data Availability

All data is contained within the article.

## References

[1] Barcena de Arellano M.L., Mechsner S. (2014). The peritoneum—An important factor for pathogenesis and pain generation in endometriosis. J. Mol. Med..

[2] Kennedy S., Bergqvist A., Chapron C., D’Hooghe T., Dunselman G., Greb R., Hummelshoj L., Prentice A., Saridogan E., Endometriosis E.S. (2005). ESHRE guideline for the diagnosis and treatment of endometriosis. Hum. Reprod..

[3] Kodaman P.H. (2015). Current strategies for endometriosis management. Obstet. Gynecol. Clin. North Am..

[4] Hey-Cunningham A.J., Peters K.M., Zevallos H.B., Berbic M., Markham R., Fraser I.S. (2013). Angiogenesis, lymphangiogenesis and neurogenesis in endometriosis. Front. Biosci..

[5] Riemma G., Lagana A.S., Schiattarella A., Garzon S., Cobellis L., Autiero R., Licciardi F., Della Corte L., La Verde M., De Franciscis P. (2020). Ion channels in the pathogenesis of endometriosis: A cutting-edge point of view. Int. J. Mol. Sci..

[6] Lagana A.S., Garzon S., Gotte M., Vigano P., Franchi M., Ghezzi F., Martin D.C. (2019). The pathogenesis of endometriosis: Molecular and cell biology insights. Int. J. Mol. Sci..

[7] Klein S., D’Hooghe T., Meuleman C., Dirksen C., Dunselman G., Simoens S. (2014). What is the societal burden of endometriosis-associated symptoms? A prospective Belgian study. Reprod. Biomed. Online.

[8] De Graaff A.A., D’Hooghe T.M., Dunselman G.A., Dirksen C.D., Hummelshoj L., Consortium W.E., Simoens S. (2013). The significant effect of endometriosis on physical, mental and social wellbeing: Results from an international cross-sectional survey. Hum. Reprod..

[9] Abbott J.A., Hawe J., Clayton R.D., Garry R. (2003). The effects and effectiveness of laparoscopic excision of endometriosis: A prospective study with 2–5 year follow-up. Hum. Reprod..

[10] Sibiude J., Santulli P., Marcellin L., Borghese B., Dousset B., Chapron C. (2014). Association of history of surgery for endometriosis with severity of deeply infiltrating endometriosis. Obstet. Gynecol..

[11] Nnoaham K.E., Hummelshoj L., Webster P., d’Hooghe T., de Cicco Nardone F., de Cicco Nardone C., Jenkinson C., Kennedy S.H., Zondervan K.T., World Endometriosis Research Foundation Global Study of Women’s Health (2011). Impact of endometriosis on quality of life and work productivity: A multicenter study across ten countries. Fertil. Steril..

[12] Wykes C.B., Clark T.J., Khan K.S. (2004). Accuracy of laparoscopy in the diagnosis of endometriosis: A systematic quantitative review. BJOG.

[13] Falcone T. (2017). Clinical management of endometriosis. Semin. Reprod. Med..

[14] Duffy J.M., Arambage K., Correa F.J., Olive D., Farquhar C., Garry R., Barlow D.H., Jacobson T.Z. (2014). Laparoscopic surgery for endometriosis. Cochrane Database Syst. Rev..

[15] Dunselman G.A., Vermeulen N., Becker C., Calhaz-Jorge C., D’Hooghe T., De Bie B., Heikinheimo O., Horne A.W., Kiesel L., Nap A. (2014). ESHRE guideline: Management of women with endometriosis. Hum. Reprod..

[16] Shakiba K., Bena J.F., McGill K.M., Minger J., Falcone T. (2008). Surgical treatment of endometriosis: A 7-year follow-up on the requirement for further surgery. Obstet. Gynecol..

[17] Vercellini P., Barbara G., Abbiati A., Somigliana E., Vigano P., Fedele L. (2009). Repetitive surgery for recurrent symptomatic endometriosis: What to do?. Eur. J. Obstet. Gynecol. Reprod. Biol..

[18] Vercellini P., Crosignani P.G., Abbiati A., Somigliana E., Vigano P., Fedele L. (2009). The effect of surgery for symptomatic endometriosis: The other side of the story. Hum. Reprod. Update.

[19] Overton C., McMillan L., Shaw R.W., Koh C. (2007). Atlas of Endometriosis.

[20] Al-Taher M., Hsien S., Schols R.M., Hanegem N.V., Bouvy N.D., Dunselman G.A.J., Stassen L.P.S. (2018). Intraoperative enhanced imaging for detection of endometriosis: A systematic review of the literature. Eur. J. Obstet. Gynecol. Reprod. Biol..

[21] Guan X., Nguyen M.T., Walsh T.M., Kelly B. (2016). Robotic single-site endometriosis resection using firefly technology. J. Minim. Invasive Gynecol..

[22] Osayi S.N., Wendling M.R., Drosdeck J.M., Chaudhry U.I., Perry K.A., Noria S.F., Mikami D.J., Needleman B.J., Muscarella P., Abdel-Rasoul M. (2015). Near-infrared fluorescent cholangiography facilitates identification of biliary anatomy during laparoscopic cholecystectomy. Surg. Endosc..

[23] van den Bos J., Al-Taher M., Schols R.M., van Kuijk S., Bouvy N.D., Stassen L.P.S. (2018). Near-infrared fluorescence imaging for real-time intraoperative guidance in anastomotic colorectal surgery: A systematic review of literature. J. Laparoendosc. Adv. Surg. Tech. A.

[24] Levey K.A. (2014). Use of fluorescence imaging technology to identify peritoneal endometriosis: A case report of new technology. Surg. Laparosc. Endosc. Percutan. Tech..

[25] Bar-Shavit Y., Jaillet L., Chauvet P., Canis M., Bourdel N. (2018). Use of indocyanine green in endometriosis surgery. Fertil. Steril..

[26] Cosentino F., Vizzielli G., Turco L.C., Fagotti A., Cianci S., Vargiu V., Zannoni G.F., Ferrandina G., Scambia G. (2018). Near-infrared imaging with indocyanine green for detection of endometriosis lesions (Gre-endo trial): A pilot study. J. Minim. Invasive Gynecol..

[27] Alander J.T., Kaartinen I., Laakso A., Patila T., Spillmann T., Tuchin V.V., Venermo M., Valisuo P. (2012). A review of indocyanine green fluorescent imaging in surgery. Int. J. Biomed. Imaging.

[28] Bishop P.A., Herron R.L. (2015). Use and misuse of the likert item responses and other ordinal measures. Int. J. Exerc. Sci..

[29] (1997). Revised American Society for reproductive medicine classification of endometriosis: 1996. Fertil. Steril..

[30] Vlek S.L., Lier M.C., Ankersmit M., Ket J.C., Dekker J.J., Mijatovic V., Tuynman J.B. (2016). Laparoscopic imaging techniques in endometriosis therapy: A systematic review. J. Minim. Invasive Gynecol..

[31] Zapardiel I., Alvarez J., Barahona M., Barri P., Boldo A., Bresco P., Gasca I., Jaunarena I., Kucukmetin A., Mancebo G. (2021). Utility of intraoperative fluorescence imaging in gynecologic surgery: Systematic review and consensus statement. Ann. Surg. Oncol..

[32] Lier M.C.I., Vlek S.L., Ankersmit M., van de Ven P.M., Dekker J., Bleeker M.C.G., Mijatovic V., Tuynman J.B. (2019). Comparison of enhanced laparoscopic imaging techniques in endometriosis surgery: A diagnostic accuracy study. Surg. Endosc..

[33] Siegenthaler F., Knabben L., Mohr S., Nirgianakis K., Imboden S., Mueller M.D. (2020). Visualization of endometriosis with laparoscopy and near-infrared optics with indocyanine green. Acta Obstet. Gynecol. Scand..

[34] Jayakumaran J., Pavlovic Z., Fuhrich D., Wiercinski K., Buffington C., Caceres A. (2019). Robotic single-site endometriosis resection using near-infrared fluorescence imaging with indocyanine green: A prospective case series and review of literature. J. Robot. Surg..

[35] You W.K., Yotsumoto F., Sakimura K., Adams R.H., Stallcup W.B. (2014). NG2 proteoglycan promotes tumor vascularization via integrin-dependent effects on pericyte function. Angiogenesis.

